# Standing on the Shoulder of Power, Representation and Relational Trust; A Response to Recent Commentaries

**DOI:** 10.34172/ijhpm.8695

**Published:** 2024-08-13

**Authors:** Anita Kothari, Rebecca Ganann, Tiffany N. Scurr, Shannon L. Sibbald

**Affiliations:** ^1^School of Health Studies, Faculty of Health Sciences, Western University, London, ON, Canada.; ^2^Faculty of Health Science, School of Nursing, McMaster University, Hamilton, ON, Canada.; ^3^Schulich Interfaculty Program in Public Health, Schulich School of Medicine and Dentistry, Western University, London, ON, Canada.

 We appreciate the commentaries by colleagues^1-3^ to our article* Evaluating Public Participation in a Deliberative Dialogue: A Single Case Study*^4^ in which we describe a co-designed deliberative dialogue process. The dialogue was meant to partner with tenants, service providers, and municipal decision-makers to generate solutions for health-related issues associated with living in the income-geared apartment building. Our aim in this response is to build on the rich conversations initiated in the commentaries by providing context and further reflections.

 Deliberative dialogues are, by their nature, a resource intensive knowledge translation strategy^5^; our study was no exception. The trauma-informed work we set out to do was challenging given the complexity of the population and their experiences. An extensive community health profile with input and interpretation from a broad representation of tenants had been developed before the dialogue.^6^ This set the foundation, ie, the priorities and the narrative for the dialogue itself, at which we wanted to collaboratively come up with actionable recommendations. Tenants self-selected to participate in the dialogue (inclusion criteria included familiarity with the needs and concerns of others in the building), and we invited service providers who worked regularly with more isolated tenants. This approach aimed to ensure a range of tenant perspectives, including those isolated and less engaged in supports available in the building, ie, those less likely to self-select. Below are our reflections on two tensions highlighted in the commentaries and a third, new tension.

## Power

 Commentators rightly raised the issue of managing and sharing power within co-production and engagement approaches. Power is perceived as an individual’s ability to achieve their own will, even against the resistance of others.^7^ Scholars have advanced theoretical developments about power but there is limited empirical evidence in the health literature that extensive thought has been given to power in project teams. Recommendations about how to manage power include things such as paying attention to representation (are the right people involved?), reflexivity (have study decisions been continually and explicitly examined?) and rigour (have the interpretations of knowledge users’ interests and perspectives been appropriately reflected?).^8^ While we made various attempts to navigate power to ensure transparency and minimize problematic power imbalances,^9^ we reflect on some aspects of power that shaped the event.

 For example, we learned from tenants that a strength of our approach was that tenants were in the space where decision-making was happening. Previously, they were engaged in a way perceived as separate from other stakeholders – as if they were at the “kids’ table” at a party – and then their input was taken to another group for further consideration. By including them in the same room and small group discussions as the other stakeholders, tenants’ views were shared in their own voices and contributed to bi-directional dialogue which was responsive to the range of views in the room. We feel that by transparently and intentionally prioritizing tenants’ commonly marginalized voices, we allowed more sharing of power and lent credibility to the dialogue process.

 At the same time, some professional stakeholders reported relinquishing their perceived power for the sake of the process. Many intentionally remained silent during segments of the discussion to allow tenants to tell their stories and provide input on the possible solutions to priority issues facing the community. “I’m here to learn” someone told us; some clinicians felt that the tenant voices needed to be prioritized.

 From these and other experiences we learned that power may not be “balanced” like a teetertotter. Someone is losing and someone is winning, and true balance may be unattainable for a long period of time. Instead, our team has turned to the concepts of fairness and inclusion in the process as a foundation for credibility and legitimacy.

## Representation

 The second tension raised by commentators revolved around representation. There were multiple ways we hoped to support feedback, such as through regular Steering Committee and Core Working Group meetings, and open invitations to participate in the dialogue. However, we encountered criticism from some tenants about who sat on the Core Working Group (eg, questions about whether these tenants could speak for everyone in the building, their ability to share power with other tenants), reflecting feelings of misrepresentation. The dialogue itself presented other forms of exclusion, such as non-English speakers and those who worked during the day. Overall, as is typical in research, we cannot claim to have had a representative sample of participants. However, we were satisfied with the diversity of experiences and challenges captured, aiming to refine the proposed solutions in the context of the tenants’ daily lives as one step of a multi-phased approach.

 With further reflection, we offer a cautionary message around the idea of achieving representation. Distilling people to a handful of characteristics may not reflect the diversity of experiences that are important to understand. And how everyone *felt* they were represented and heard might be more important than how much they spoke.

## Relational Trust

 A third tension was experienced throughout the dialogue planning and implementation process. It was critical to develop relational trust in the context of historical and ongoing experiences with trauma, both at individual levels and as tenants of the rent-geared-to-income housing complex, which faced significant neighbourhood disorder.^10^ When working with the tenants, the researchers quickly faced relational trust as a tension, as the tenants expressed that they had been involved in previous research where they were “surveyed to death” and “it didn’t go anywhere. Nothing changed.” Both providers and researchers were cognizant of previous traumas and harms experienced by these tenants and made significant investments in relationship building over a prolonged period and numerous onsite visits to understand the context. In the article we have a supplementary file that provides context. Establishing these collaborative relationships required navigating tenuous relationships between tenants and providers, tenants and each other, and tenants and researchers. It required working with specific people with existing relationships, which were not always trusting and synergistic. It required overcoming previous misalignments, new ways of working together, terms of engagement, and establishing a shared vision.^11^

## Ethical issues

 Not applicable.

## Competing interests

 Authors declare that they have no competing interests.
